# How does task complexity and task difficulty affect college students’ writing performance? The mediating effect of critical thinking disposition

**DOI:** 10.1371/journal.pone.0324486

**Published:** 2025-06-04

**Authors:** Yumei Zou, Florence Kuek, Xiaoli Cheng, Wenqin Feng

**Affiliations:** 1 School of Foreign Languages, Jiangxi Agricultural University, Nanchang, Jiangxi, China; 2 Faculty of Arts and Social Sciences, Universiti Malaya, Kuala Lumpur, Malaysia; 3 Nanchang University Gongqing College, Gongqingcheng, Jiangxi, China; Golestan University, IRAN, ISLAMIC REPUBLIC OF

## Abstract

This study investigated the effects of task complexity, task difficulty, and critical thinking disposition on college students’ writing performance. 201 participants were selected via probability sampling. Each completed three writing tasks of varying complexity, resulting in a corpus of 603 compositions. A follow-up questionnaire assessed task difficulty and critical thinking disposition. Results showed that both task complexity and difficulty significantly affected writing performance. Specifically, four sub-constructs of critical thinking disposition (analyticity, systematicity, inquisitiveness, and cognitive maturity) were positively associated with writing performance and mediated the relationship between task difficulty and writing performance. Structural and mediation models were established using Smart PLS to illustrate these relationships. This study contributes to the growing field of cognitive-oriented and affective-related research by exploring the cognitive and affective factors influencing writing and provides practical recommendations for writing assessment and pedagogy.

## Introduction

Writing is a crucial indicator of language proficiency. Previous studies have indicated that learners’ writing performance is influenced by numerous factors, including task design and assessment type [1,2]. Task characteristic plays a significant role in language assessment, contributing to validation and evaluation of tests [3,4].

Past research has highlighted that task complexity significantly impacts writing performance [3,4]. As Long (2014) argues, a task-based syllabus should present learners with challenges that progress in complexity, ultimately approaching real-world demands [5]. Similarly, task difficulty has been identified as a key factor influencing performance [6]. Difficult tasks can lead to anxiety and frustration due to increased cognitive load, while easier tasks foster confidence and ease [7]. Affective factors such as motivation, confidence, competence, and anxiety are also essential predictors of writing performance [6,8].

While many studies have examined task complexity and difficulty objectively, few have investigated these factors subjectively – from a learner’s perspective. Understanding how individuals perceive task complexity and difficulty is crucial, especially in the context of formative and summative assessment. Furthermore, there is growing empirical and theoretical support from previous studies on the close relationship between writing and critical thinking [9,10]. However, the specific impact of critical thinking disposition on writing performance, particularly in the context of college English instruction in China, remains under-researched.

To this end, the current study addresses this gap by examining the relationship between task complexity, task difficulty, and critical thinking disposition on college students’ English writing performance in China. The investigation explores how learners perceive task complexity and difficulty in national college English writing tests and aims to highlight the importance of cultivating critical thinking disposition in writing instruction. National college English writing tests are widely used in China to assess students’ writing proficiency and guide teaching practices. These tests are chosen as the focus of this study because of their significant impact on educational outcomes. By investigating these interrelationships, this study aims to provide valuable insights for test designers and language instructors, ultimately contributing to more effective writing pedagogy and assessment. This study aims to address the following research questions: (1) What are the effects of task complexity, task difficulty on students’ writing performance? (2) Is there a significant relationship between critical thinking disposition and writing performance? (3) Is there a mediation effect of critical thinking disposition between the relationship of task difficulty and writing performance?

## Literature review

### Theories underpinning the study

Robinson’s Cognition Hypothesis (2001) provided the theoretical basis for the current study. This model, which is also called the Triadic Componential Framework, established the connection between task complexity, task difficulty, and writing performance. In this model, there exist three components in a task, that is task complexity, task condition, and task difficulty [7]. These three components developed different aspects of tasks, cognitive factor, interaction factor, and learner factor, respectively. Task complexity highlighted the internal structure of the task, that is, the cognitive factor. In contrast to task complexity, task difficulty was determined by learners’ ratings of the tasks in relation to affective factors, like motivation, anxiety, and ability factors, like intelligence, working memory, and language aptitude. An affective variable was changeable, while the ability factor was stable and fixed.

Additionally, motivational theory [11] has shown a correlation between critical thinking disposition and writing performance, which argues that the learners’ eagerness, willingness, and desire to write affect their achievement in writing. Learners’ behavior and achievement are partially attributed to their motivation to be involved in the task. Motivational theory suggests that students attain what they value by showing a willingness to engage in a task successfully. Students’ ability to write successfully may be enhanced by aligning with these seven dispositions of critical thinking. This argument was backed up by the leading figure Freud who has dominated western psychology for the past century. As with other philosophers, he asserted that the mainsprings of conduct could be hidden from conscious awareness [12].

### Task complexity, task difficulty and writing performance

This study is grounded in Robinson’s Triadic Componential Framework. Task complexity and task difficulty are distinct yet interrelated concepts. While task complexity refers to the cognitive demands inherent in a task itself, task difficulty reflects the learner’s perception of those demands [7]. Although research on the cognitive aspects of writing is growing [13], further exploration is necessary, particularly regarding task complexity and difficulty.

The concept of task complexity stems from task-based language teaching (TBLT) and the notion that tasks can be sequenced based on their cognitive demands [6,13–15]. Higher complexity generally implies greater cognitive effort. Numerous studies have investigated the impact of task complexity on language performance, often guided by prominent frameworks in second language acquisition and TBLT.

Task difficulty, on the other hand, encompasses both affective variables, such as confidence, motivation, and anxiety, as well as ability variables, such as intelligence, aptitude, and cognitive style [6]. In EFL writing, research has highlighted the influence of affective factors on writing processes and outcomes [8,16,17]. For instance, an empirical study that examined 100 Iraqi undergraduate English majors from two Iraqi public universities found a strong correlation between anxiety and writing performance, with higher anxiety often leading to poorer outcomes [8].

While task complexity and difficulty have been extensively studied, there are still challenges in defining and measuring these constructs. Some researchers argue that current approaches conflate task characteristics with learner ability, leading to inconsistent results [18]. Therefore, a more nuanced understanding of how task complexity and difficulty interact with individual learner characteristics is needed.

Despite these challenges, tasks remain a central focus in language teaching and syllabus design [14]. Researchers largely agree that task complexity is a crucial criterion for sequencing tasks, with gradual progression from less to more complex tasks mirroring real-world demands [3,7,14,15,19].

### Critical thinking disposition and writing performance

The relationship between critical thinking and writing is well established [20]. Critical thinking (CT) is closely linked to writing performance [10,21], and research has demonstrated its impact on EFL (English as a Foreign Language) college students’ writing [6,9,21]. Both constructs are complex, socially constructed, contextually situated, and involve higher-order thinking skills [22,23]. Writing fosters critical thinking, and a disposition towards critical thinking, in turn, facilitates effective writing [24,25].

Critical thinking disposition, an important non-intellectual aspect of CT ability [17], encompasses traits such as truth-seeking, open-mindedness, analyticity, systematicity, self-confidence, inquisitiveness, and cognitive maturity [18]. These scales are essential for successful college-level writing [9]. Studies have also found that the involvement of critical thinking disposition strategies in the writing process could result in higher-quality writing compositions [26].

The link between critical thinking disposition and writing performance can be explained through Motivational Theory [27], which posits that a positive disposition towards a task enhances engagement and achievement. Kellogg (1994) echoed this notion, with the concept of creative flow, which describes the tendency of the author to fully engage with the task of writing. Similarly, Hayes (2000, 2006) considered personality in the context of “writer’s motives/effect,” modifying his original cognitive model, including the writer’s belief, motivation, tendency, targets, and perceptions of required efforts [28].

Critical thinking disposition, distinguished from critical thinking abilities, involves the interplay of cognitive, emotional, and social factors [24]. Critical thinking disposition reflects a willingness to engage in using their critical thinking abilities and persist when faced with a problem. Thus, students’ performance in problem-solving is closely related to their engagement of critical thinking disposition.

Writing is a complex process involving cognitive and affective factors, requiring learners to engage various cognitive skills in response to different tasks [29]. As stated by Lubart (1999), creativity is defined as the ability to come up with new ideas and original products. While research has explored task types and the quality of language production, with a focus on text quality, less attention has been given to the impact of individual cognitive differences on writing outcomes.

Critical thinking, a key cognitive skill in writing, involves six aspects, interpretation, analysis, evaluation, inference, explanation, and self-regulation. Experts emphasize the importance of both critical thinking skills and dispositions for effective critical thinking [30,31]. Given the significant influence of critical thinking disposition on writing [32], fostering students’ comprehension of cognitive levels and encouraging creativity within a conducive classroom environment are crucial for task-based learning [33,34].

While students may possess critical thinking skills, they may not apply them effectively, particularly when faced with controversial questions or multiple ideas [25,35]. Therefore, critical thinking disposition encompasses not only the willingness to employ critical thinking skills but also the awareness of when they are needed in a given context. Key components of critical thinking disposition include truth-seeking, open-mindedness, analyticity, systematicity, self-confidence, inquisitiveness, and cognitive maturity [23]. In writing practice, students who effectively integrate these dispositions into their writing process are likely to achieve better outcomes.

Few studies have examined the mediating effect of critical thinking disposition on the relationship between task difficulty and college students’ writing performance. To address this gap, the current study tested a structural equation model using Smart PLS-SEM, which hypothesizes that critical thinking disposition has a mediation effect on the relationship between task difficulty and college students’ writing performance. This insight is crucial for designing effective writing instruction and assessment strategies that support students in developing higher-order thinking skills and improving their overall academic performance.

## Methods

### Participant recruitment

The participants in this study were 278 undergraduate students from a comprehensive, open-enrollment college in Jiangxi Province, China. All participants had previously taken and passed the College English Test Band Four (CET-4), indicating their relevant writing experience and proficiency in English. Participants were selected using probability sampling to ensure a representative and homogeneous sample with similar ages and English learning experiences. This selection criterion helped focus the study on individuals with a common background in CET-4 writing tasks, aligning with the research objectives. While 278 participants were initially recruited between May 1^st^ and June 1^st^, 2023,77 did not complete all three tasks, leaving a final sample of 201.

All participants provided verbal informed consent, which was obtained after a thorough explanation of the study’s aims, procedures, potential risks, and benefits.

### Ethical considerations

This study was approved by the Jiangxi Agricultural University (No.: JXAUSFL202301), where the study was conducted. Ethical approval was obtained for this study. The relevant approval information was detailed in the manuscript.

### Task complexity description

Over three consecutive weeks, the initial sample of 278 college students completed three writing tasks of varying complexity (see Table 1).

**Table 1:** Three writing tasks used in this study.

Tasks	Task 1	Task 2	Task 3
Writing tasks	Write a short essay about a classmate of yours who has influenced you most	Start your essay with a brief account of the increasing use of the mobile phone in people’s lives and then explain the consequences of overusing it.	Write a short essay on *the use of robots.* Try to imagine what will happen when more and more robots take the place of human beings in the industry as well as in people’s daily lives.
Genre	narration	Argumentation	argumentation
Theme	Personal experience	Daily technology	Frontier technology
Reasoning demands	--	+	++
Previous knowledge	+	+	--
Task complexity	least-complex	mid-complex	most-complex

Task complexity was classified based on Robinson’s Triadic Componential Framework, focusing on the cognitive demands of the tasks. This classification, in relation to prior knowledge, was supported by Skehan (1996), who argued that familiarity with a topic influences cognitive load. That is, the lower the degree of familiarity, the heavier the cognitive burden for the participants and consequently, the more complex the task [15]. Task 1 was the least complex, Task 2 was moderately complex, and Task 3 was the most complex.

### Writing performance evaluation

To ensure a comprehensive evaluation of student writing, this study employed a multifaceted assessment framework focusing on four key dimensions: complexity, accuracy, lexical diversity, and fluency (CALF). These dimensions, commonly used in task-based writing research, allowed for a nuanced analysis of various aspects of writing performance. Specifically, each essay was analyzed on its syntactic complexity, grammatical accuracy, vocabulary range, and overall fluency. Three experienced English teachers with master’s degrees applied this framework to each writing sample, ensuring a comprehensive and nuanced evaluation. To ensure the reliability and consistency of the evaluation, raters were trained prior to the assessment. During the training, they were provided with clear scoring criteria and detailed guidelines for each dimension (Complexity, Accuracy, Lexical Diversity, and Fluency). Raters then participated in a pilot scoring session to standardize their assessments and refine the criteria. The final evaluation was conducted holistically, integrating the four dimensions into an overall score. To minimize potential bias, the researcher and classroom instructor were not involved in the evaluation process. Reliability statistics indicated a high level of inter-rater agreement, with internal consistency ranging from 0.900 to 0.946, reflecting excellent agreement among the raters in their judgments.

### Instruments

This study utilized a questionnaire to measure task difficulty and critical thinking disposition (see Table 2).

**Table 2:** Research instruments in the current study.

Variables	Instruments	Adapted from
Task difficulty	Task difficulty scale	Robinson (2001)
Critical thinking disposition	CTDI-CV questionnaire	Peng, et al. (2004)

Following the writing activity, the students completed the questionnaire assessing task difficulty [7] and critical thinking disposition [18]. The validity and reliability of both instruments were assessed using Smart-PLS, with results presented in Tables 3–5.

**Table 3:** Construct reliability and validity of task difficulty.

	Cronbach’s Alpha	rho_A	Composite Reliability	Average Variance Extracted (AVE)
Task difficulty (Task 1)	0.917	0.917	0.938	0.750
Task difficulty (Task 2)	0.910	0.910	0.933	0.735
Task difficulty (Task 3)	0.910	0.911	0.933	0.736

**Table 4:** Construct reliability and validity of critical thinking disposition.

	Cronbach’s Alpha	rho_A	Composite Reliability	Average Variance Extracted (AVE)
truth-seeking	0.859	0.868	0.899	0.859
open-mindedness	0.721	0.721	0.843	0.721
analyticity	0.896	0.899	0.917	0.896
systematicity	0.850	0.861	0.888	0.850
self-confidence	0.723	0.732	0.844	0.723
inquisitiveness	0.912	0.915	0.927	0.912
cognitive maturity	0.900	0.905	0.919	0.900

**Table 5:** Discriminant validity of critical thinking disposition.

	analyticity	cognitive maturity	inquisitiveness	open-mindedness	self-confidence	systematicity	truth-seeking
analyticity	0.611						
cognitive maturity	0.554	0.268					
inquisitiveness	0.556	0.302	0.156				
openminded	0.444	0.121	0.102	0.260			
self-confidence	0.409	0.107	0.085	0.094	0.124		
systematicity	0.438	0.195	0.192	0.175	0.087	0.104	
truth-seeking	0.473	0.100	0.090	0.105	0.198	0.671	0.105

Task difficulty was evaluated based on Robinson’s [7] five aspects: overall perception of task difficulty, ratings of stress, perceived ability to complete the task, interest in task content, and motivation to complete these and other similar tasks. The measurement model for task difficulty was assessed using PLS Algorithm.

The critical thinking disposition questionnaire involved seven dimensions, comprising 58 items after pilot study testing. Items with outer loading below 0.7 were excluded in the final study. The final reliability and validity results are presented in Table 4.

As shown in the table above, the Cronbach’s alpha, composite reliability, and AVE values met the required threshold, indicating high reliability and validity for the instruments used in this study. Specifically, Cronbach’s alpha values for the critical thinking disposition scale ranged from 0.721 to 0.912, exceeding the recommended value of 0.7. Similarly, composite reliability values (0.843 to 0.927) and AVE value (0.721 to 0.912) surpassed their respective thresholds of 0.7 and 0.5.

## Results

### Effects of task complexity, task difficulty and writing performance

To analyze the impact of task complexity on writing performance, descriptive analysis, Levene’s test, one-way ANOVA, and post-hoc tests were conducted. The results are presented in the following tables (Tables 6–9).

**Table 6:** Descriptive statistics of writing performance among three writing tasks.

tasks	N	mean	Std. Deviation	std. Error	minimum	maximum
1	201	10.03	0.98	0.07	5.33	12.67
2	201	10.80	1.03	0.07	8.00	13.67
3	201	10.72	0.98	0.07	8.00	13.33
total	603	10.52	1.05	0.04	5.33	13.67

**Table 7:** One-way ANOVA analysis of writing performance.

Writing performance	Sum of Squares	df	Mean Square	F	Sig.
Between Groups	72.231	2	36.116	36.135	.000
Within Groups	599.679	600	.999		
Total	671.910	602			

**Table 8:** Path coefficient of task difficulty.

Path Coefficient	Original Sample (O)	T Statistics (|O/STDEV|)	P Values
Task difficulty 1 - > WP1	0.386	6.821	0.000
Task difficulty 2 - > WP 2	0.306	4.766	0.000
Task difficulty 3 - > WP 3	0.348	6.478	0.000

**Table 9:** Predictive powers of task difficulty on writing performance.

	R²	T Statistics	P Values	Q²	SRMR
Task 1 scores	0.149	3.357	0.001	0.115	0.052
Task 2 scores	0.094	2.335	0.02	0.077	0.043
Task 3 scores	0.121	3.176	0.001	0.104	0.048

Table 6 presents the descriptive statistics for the 603 essays, completed by 201 participants across three writing tasks. Task 2 exhibited the highest mean score (10.80, SD = 1.03), following by Task 3 (10.72, SD = 0.98), and then Task 1 (10.03, SD = 0.98). Levene’s Test for Equality of Variance (p = 0.626, p > 0.05) indicated homogeneity of variance across the three tasks.

A one-way ANOVA revealed a significant difference in the mean writing performance across the three tasks, F (2, 600) – 36.135, p < .01).

Post-hoc tests using the Tukey HSD and LSD methods revealed significant difference in writing performance between Task 1 and both Task 2 (p < .001) and Task 3 (p < .001). However, no significant difference was found between Task 2 and Task 3 (p > .05).

Table 8 depicts the path coefficient results of task difficulty and writing performance among three writing tasks. The results showed that path coefficients in writing task 1 model (ß = 0.386, t = 6.821, p < 0.001), task 2 model (ß = 0.306, t = 4.766, p < 0.001), and task 3 model (ß = 0.348, t = 6.478, p < 0.001) were statistically significant, suggesting that task difficulty significantly predicted writing performance across all three tasks. This indicates that students with higher affective and ability skills achieved higher writing scores.

The results in Table 9 reveals that the R² values for Task 1 and Task 3 models exceeded 0.1, which is the threshold strength to determine each structural path for the dependent variable [25]. Thus, the predictive capability of task difficulty on writing performance in the two models is established. The Q² values for these models (0.115 and 0.104, respectively) were also larger than zero, demonstrating predictive relevance. Furthermore, the SRMR values (0.052 and 0.058) were below the 0.1 threshold, indicating the acceptable model fit.

In contrast to Tasks 1 and 3, the Task 2 model showed an R² value of 0.094, slightly below the 0.1 threshold, suggesting weaker predictive power for task difficulty in this model. However, the model demonstrated predictive relevance of the endogenous constructs. A Q² above 0 shows that the model has predictive relevance [36]. Additionally, the SRMR value of 0.042 indicated acceptable model fit [36].

### Relationship between critical thinking disposition and writing performance

A structural model was established to assess the causal relationships between critical thinking disposition and writing performance. Model fit was evaluated using the significance of path coefficients and the R² value for the dependent variable (Brones Penalver et al., 2018). The value for R² value should be equal to or over 0.1 [25].

Table 10 shows the relationship between the seven dimensions of critical thinking disposition and writing performance (WP) for Task 1. Four dimensions had significant positive impacts on WP: analyticity (ß = 0.266, t = 4.813, p < .001), cognitive maturity (ß = 0.203 t = 4.025, p < .001), systematicity (ß = 0.293, t = 5.331, p < 0.001), three of them (truth-seeking, open-mindedness, self-confidence) did not have significant impacts on writing performance, while the other four of critical thinking disposition (analyticity, cognitive maturity, inquisitiveness, and systematicity) had significant impacts on writing performance (WP) of writing task 1. Specifically, had a significant impact on writing performance. By contrast, open-mindedness had a negative relationship with writing performance (ß = −0.061, t = 0.887, p = 0.375, p > 0.05), while self-confidence (ß = 0.12, t = 1.414, p = 0.157, p > 0.05), truth-seeking (ß = 0.016, t = 0.286, p = 0.775, p > 0.05) revealed no significant contribution to writing performance, indicating these three dimensions were not significantly related to writing performance. This study’s 5000 subsamples also generated 95% confidence intervals, as shown in Table 10. A confidence interval different from Zero indicated a significant relationship.

**Table 10:** Path coefficient of critical thinking disposition (Task 1).

Path coefficient	Original Sample	T Statistics	P Values	Confidence intervals
analyticity - > WP	0.268	**4.828**	**0.000**	[0.160; 0.375]
cognitive maturity - > WP	0.203	**4.176**	**0.000**	[0.109; 0.300]
inquisitiveness - > WP	0.308	**5.787**	**0.000**	[0.210; 0.415]
open mindedness - > WP	−0.061	0.887	0.375	[-0.197; 0.056]
self-confidence - > WP	0.12	1.414	0.157	[-0.105; 0.240]
systematicity - > WP	0.293	**5.331**	**0.000**	[0.181; 0.396]
truth-seeking - > WP	0.016	0.286	0.775	[-0.115; 0.110]

Similarly, as shown in Tables 11 and 12, path coefficient values of Task 2 and Task 3 showed that four dimensions of critical thinking disposition (analyticity, cognitive maturity, inquisitiveness, and systematicity) had significant impacts on writing performance. The other three dimensions (open-mindedness, self-confidence, and truth-seeking) did not have significant impacts on writing performance.

**Table 11:** Path coefficient of critical thinking disposition (Task 2).

Path coefficient	Original Sample	T Statistics	P Values	Confidence intervals
analyticity - > WP	0.266	**5.043**	**0.000**	[0.166; 0.373]
cognitive maturity - > WP	0.307	**6.011**	**0.000**	[0.204; 0.405]
inquisitiveness - > WP	0.281	**6.261**	**0.000**	[0.188; 0.363]
open-mindedness - > WP	0.095	1.746	0.081	[-0.018; 0.197]
self-confidence - > WP	−0.096	1.208	0.227	[-0.225; 0.077]
systematicity - > WP	0.258	**5.618**	**0.000**	[0.168; 0.345]
truth-seeking - > WP	0.119	1.794	0.073	[-0.020; 0.241]

**Table 12:** Path coefficient of critical thinking disposition (Task 3).

Path coefficient	Original Sample	T Statistics	P Values	Confidence intervals
analyticity - > WP	0.298	**5.103**	**0.000**	[0.177;0.412]
cognitive maturity - > WP	0.235	**4.889**	**0.000**	[0.139; 0.331]
inquisitiveness - > WP	0.334	**6.177**	**0.000**	[0.236; 0.441]
open-mindedness - > WP	0.014	0.203	0.839	[-0.178; 0.112]
self-confidence - > WP	0.080	0.693	0.488	−0.137; 0.240]
systematicity - > WP	0.209	**3.583**	**0.000**	[0.090; 0.316]
truth-seeking - > WP	0.090	1.303	0.193	[-0.161; 0.187]

The structural model was further assessed by the strength of each structural path determined by the R² value for the dependent variable and Q² for the establishment of the predictive relevance of the endogenous constructs as well as the SRMR value [36]. The literature said that R² values should be equal to or over 0.1 [25].

The results in Table 13 revealed that all R² values are over 0.2. Hence, the predictive capability is established. Further, Q² establishes the predictive relevance of the endogenous constructs. A Q² above 0 indicates that the model has predictive relevance. The results show that there is significance in the prediction of the constructs. Furthermore, the model fit was assessed using SRMR. The value of SRMR was 0.072. This is below the required value of 0.10, indicating an acceptable model fit [36].

**Table 13:** Goodness of model fit (Critical thinking disposition and writing performance).

	R²	T Statistics	P Values	Q²	SRMR
Task 1 scores	0.446	8.353	0.000	0.371	0.059
Task 2 scores	0.535	12.001	0.000	0.452	0.057
Task 3 scores	0.491	11.35	0.000	0.417	0.067

According to Hair et al. (2016), for structural model assessment, R² values of 0.75, 0.50, and 0.25 are considered substantial, moderate, and weak. Table 13 revealed that R² values of structural models of the three writing tasks range from 0.446 to 0.535, and the predictive capability is established, indicating a moderate predictive capability of critical thinking disposition on writing performance. Specifically, the Task 2 model showed the strongest predictive capability, suggesting that critical thinking disposition has the greatest impact on writing performance in this task. In addition, Q² values larger than zero are considered meaningful. Higher than 0.025 and 0.50 depict small, medium, and large predictive accuracy of the PLS path model. Q² of the three models were 0.371, 0.452, and 0.417, respectively, suggesting that the PLS path model has large predictive relevance. There is significance in the prediction of critical thinking disposition. Finally, values of SRMR were 0.059, 0.057, and 0.067, which is below the threshold value of 0.10, indicating acceptable model fit.

### Mediation effect of critical thinking disposition on the relationship between task difficulty and writing performance

Mediation analysis was performed to assess the mediating role of critical thinking disposition (analyticity, systematicity, inquisitiveness, and cognitive maturity) on the relationship between task difficulty and writing performance. The core characteristic of a mediating effect (i.e., indirect effect or mediation) is that it involves a third variable that plays an intermediate role in the relationship between the independent and dependent variables. [37]. A mediation analysis model was performed to assess the mediating role of critical thinking disposition (CT) on the relationship between task difficulty (TD) and writing performance (WP). This analysis examined whether critical thinking disposition acts as an intermediary factor, influencing the relationship between task difficulty and writing performance. The results are shown in Tables 14–16.

**Table 14:** Mediation analysis of critical thinking disposition (Task 1).

Task 1	Path coefficient	Original Sample	T value	P-value	BI (2.5%;97.5%
Path coefficient	CT- > WP	0.605	12.81	0.000	0.495; 0.689
	TD- > CTD	0.428	6.703	0.000	0.282; 0.538
	TD- > WP	0.124	2.043	0.041	0.004; 0.243
Total effect	CT- > WP	0.605	12.81	0.000	0.495; 0.689
	TD- > CTD	0.428	6.703	0.000	0.282; 0.538
	TD- > WP	0.383	6.43	0.000	0.250;0.490
Indirect effect	TD- > CTD- > WP	0.259	5.396	0.000	0.161; 0.347

**Table 15:** Mediation analysis of critical thinking disposition (Task 2).

Task 2	Path coefficient	Original Sample (O)	T value	P-value	BI (2.5%;97.5%
Path coefficient	CTD- > WP	0.697	17.836	0.000	0.613; 0.766
	TD- > CTD	0.392	5.807	0.000	0.236; 0.508
	TD- > WP	0.032	0.642	0.521	0.066; 0.129
Total effect	CTD- > WP	0.697	17.836	0.000	0.613; 0.766
	TD- > CTD	0.392	5.807	0.000	0.236; 0.508
	TD- > WP	0.305	4.743	0.000	0.163; 0.420
Indirect effect	TD- > CTD- > WP	0.273	5.372	0.000	0.165; 0.367

**Table 16:** Mediation analysis of critical thinking disposition (Task 3).

Task 3	Path coefficient	Original Sample (O)	T value	P-value	BI (2.5%;97.5%
Path coefficient	CT- > WP	0.65	16.018	0.000	0.559; 0.719
	TD- > CT	0.371	5.972	0.000	0.233; 0.480
	TD- > WP	0.102	2.03	0.042	0.005; 0.200
Total effect	CT- > WP	0.65	15.861	0.000	0.559; 0.719
	TD- > CT	0.371	5.963	0.000	0.233; 0.480
	TD- > WP	0.343	6.197	0.000	0.221; 0.445
Indirect effect	TD- > CT- > WP	**0.241**	**5.564**	**0.000**	0.147; 0.320

The results of Task 1 shown in Table 14 revealed that the total effect of TD on WP was significant (ß = .383, t = 6.43, p < .001), with the presence of mediating variable critical thinking disposition, the impact of task difficulty on critical thinking disposition was still significant (ß = .259, t = 5.396, p < .001), indicating that the indirect effect of task difficulty on writing performance was partially mediated by critical thinking disposition.

**Fig 1:**
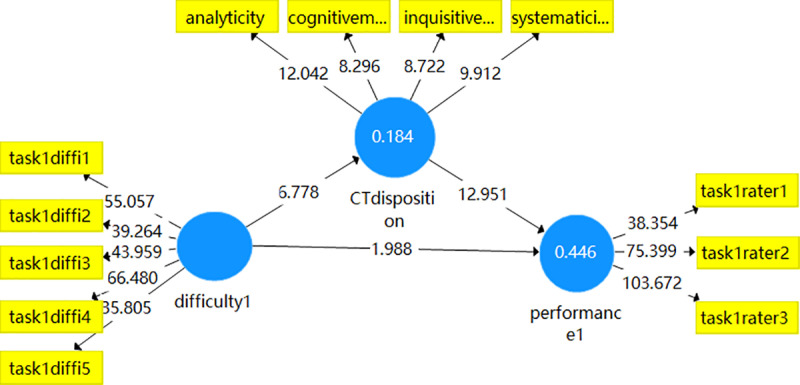
Mediation model of critical thinking disposition in writing task 1.

Additionally, the PLS mediation model (see Fig 1) displayed the significance of T statistics of path coefficient among the variables. The model indicated that task difficulty had a significant direct effect on writing performance (T > 1.96). Meanwhile, with the presence of the mediator variable, critical thinking disposition, the indirect path was still significant, meaning that the four constructs (analyticity, cognitive maturity, inquisitiveness, and systematicity) could mediate between task difficulty and writing performance.

The results of Task 2 shown in Table 15 revealed that the total effect of TD on WP was not significant (ß = .032, t = 642, p > .05), with the presence of mediating variable critical thinking disposition, the impact of task difficulty on critical thinking disposition was significant (ß = .273, t = 5.372, p < .001), indicating that the indirect effect of task difficulty on writing performance was fully mediated by critical thinking disposition.

**Fig 2:**
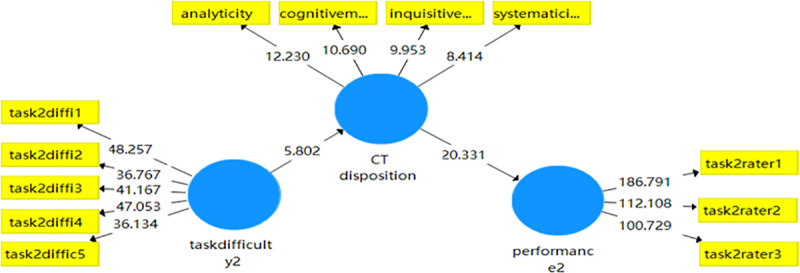
Mediation model of critical thinking disposition in writing task 2.

The Smart PLS model for Task 2 (see Fig 2) indicated that EFL learners with critical thinking disposition are more likely to regulate task difficulty, which in turn predicts increased writing performance. Path coefficient model of Task 2 showed that task difficulty in the absence of critical thinking disposition did not predict writing performance, meaning that task difficulty could only exert an impact on writing performance through critical thinking disposition. This highlights the crucial role of critical thinking disposition in this model when the task required more cognitive reasoning demand. The results suggested that analyticity, systematicity, inquisitiveness, and cognitive maturity contributed more to process the tasks that require learners’ more disposition in critical thinking. These findings highlight the role of reasoning and evidence in the writing process, reinforcing the link between critical thinking and writing performance

**Fig 3:**
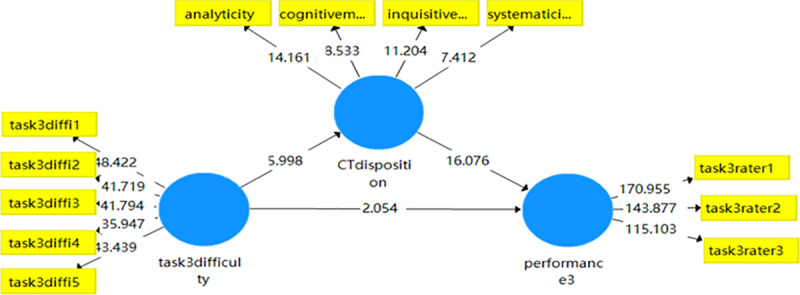
Mediation model of critical thinking disposition of task 3.

Similarly, the results of Task 3 shown in Table 16 revealed that the total effect of TD on WP was still significant (ß = .343, t = 6.197, p < .001), with the presence of mediating variable critical thinking disposition, the impact of task difficulty on critical thinking disposition was still significant (ß = .241, t = 5.506, p < .001), indicating that the indirect effect of task difficulty on writing score was partially mediated by critical thinking disposition. The PLS mediation model for Task 3 (see Fig 3) revealed the significance of the path coefficients among the variables. Specifically, the model showed that task difficulty exerted a significant direct effect on writing performance (T > 1.96). Additionally, when critical thinking disposition was included as a mediator, the indirect path remained significant. This indicates that critical thinking disposition can mediate the relationship between task difficulty and writing performance.

In summary, mediation model analysis revealed that critical thinking disposition played a mediating role between task difficulty and writing performance. However, the mediating effect of critical thinking disposition between task difficulty and writing performance varied across task types, with partial mediation observed in Tasks 1 and 3 and full mediation in Task 2.

## Discussion

### The effect of task complexity, task difficulty on writing performance

This study found that both task complexity and task difficulty significantly predicted writing performance. Notably, the predictive power of task difficulty varied across task types. Specifically, task difficulty was a stronger predictor of Task 1 (narrative) and Task 3 (descriptive) compared to Task 2 (argumentative). This finding aligns with previous research, such as that of Robinson (2001), demonstrating the influence of task characteristics on learner behavior [35,38,39]. Besides, other studies by Xing (2023) have shown that task complexity and difficulty significantly impact learners’ cognitive activities and language performance [40].

The weaker predictive power of task difficulty in Task 2 may be attributed to several factors. First, students likely had more experience with argumentative essays, reducing the influence of affective factors like motivation and confidence. In contrast, Tasks 1 and 3 presented less familiar topics and writing styles, potentially increasing the impact of anxiety and perceived difficulty on performance.

Second, familiarity with the content of Task 2 may have facilitated fluency and lexical diversity, further diminishing the role of affective factors. Conversely, the less familiar topics in Tasks 1 and 3 may have heightened the cognitive demands and the influence of perceived difficulty. Furthermore, students were allowed to use their cellphones, which assisted them to write more fluently.

These findings underscore the complex interplay between task characteristics, learners’ perception, and writing performance. While learners’ perception was not as significant in Task 2, learners’ perception of task difficulty had strong predictive capability on writing performance in Task 1 and Task 3.

### Mediation effect of critical thinking disposition

A critical thinking disposition, characterized by a willingness to expend cognitive effort in solving problems [41] interacts with affective factors associated with task difficulty to influence writing performance. This interplay aligns with Lewin’s Motivational Theory [11], which suggests that valuing and using critical thinking motivates individuals to achieve mastery.

The mediation model revealed that critical thinking disposition played a mediating role on the relationship between task difficulty and writing performance in all three writing tasks, with full mediation in Task 2 and partial mediation in Tasks 1 and 3. Path coefficient, total effect, and indirect effect were analyzed to identify the mediating effect. This indicates that task difficulty alone does not fully explain writing performance; rather, the disposition to engage in critical thinking is crucial for translating perceived difficulty into successful writing outcomes.

The varying mediation effects across tasks highlight the context-dependent nature of this relationship. In Task 2, which required more cognitive reasoning, critical thinking disposition played a full mediating role, suggesting its heightened importance in cognitively demanding tasks. The partial mediation in Tasks 1 and 3 may be attributed to several factors. Task 1, with its lower cognitive demand, may not have fully activated critical thinking dispositions. In Task 3, unfamiliarity with the topic may have limited the engagement of critical thinking skills.

The significant role of critical thinking disposition in Task 2 underscores the importance of this disposition in complex tasks. As cognitive reasoning demands increase, the engagement of critical thinking disposition also rises, leading improved writing performance. This aligns with Robinson’s argument that increased task complexity encourages learners to fully utilize their attention, resulting in better performance.

Tasks with moderate reasoning demands were facilitative factors for learners’ articulation of their voices. Thus, reasoning demanding tasks matched students’ writing habits and language proficiency. As a result, tasks that require a higher cognitive load, increases language proficiency, which in turn contributes to high-quality essays and higher writing scores.

These findings can be attributed to the connection between writing and cognition. Critical thinking disposition reflects a willingness to engage in cognitive effort during the writing process [17]. From the perspective of cognitive integration, writing alters our cognitive abilities: a task is made possible by a coordinated interaction between cerebral processes, physical processes, and manipulating written phrases [42]. Existing literature suggests that second language users must display the skills needed to process information, reason from evidence, make decisions, solve problems, self-regulate, collaborate, and learn. These requirements have created performance demands for the involvement of critical thinking disposition in writing tests [33].

The partial mediation observed in Task 1 can be attributed to its lower cognitive demand, which may not have fully engage critical thinking dispositions. In Task 3, the unfamiliarity with the topic, “robots,” may have limited the use of critical thinking skills, as suggested by previous research linking topic familiarity to critical thinking [34]. This suggests that tasks requiring more complex reasoning and drawing on familiar topics may better activate critical thinking dispositions and, consequently, influence writing performance.

In this respect, this study highlights the importance of critical thinking disposition in writing, particularly for tasks involving moderate cognitive demands and familiar topics. Such tasks can effectively activate critical thinking dispositions, leading to improve writing performance. This finding aligns with previous research emphasizing the influence of critical thinking disposition on student performance [24].

The mediating role of critical thinking disposition between task difficulty and writing performance further underscores the complex cognitive processes involved in writing. This study’s findings, along with previous research linking critical thinking disposition to problem-solving and metacognitive awareness among students, emphasize the crucial role of this disposition in achieving successful writing outcomes.

## Conclusion

Research on task-based writing from a cognitive aspect is still emerging. This study contributed to this growing field of research by exploring the effects of task complexity on EFL college students’ writing performance. Specifically, the influence of task complexity on writing performance was examined. The study found that tasks requiring reasoning stimulated higher-scoring essays, encouraging learners to utilize their full potential and attention. Tasks requiring higher reasoning demands help learners engage their cognitive potential more effectively. This has important implications for language instruction, cognitive psychology and assessment design, suggesting the need to align tasks with learners’ proficiency levels and cognitive capabilities.

Beyond cognitive influences, this study also examined how affective factors shape writing performance in task-based language assessments. By investigating the relationship between task difficulty and writing performance, this study revealed that the influence of affective factors varied depending on task characteristics.

More importantly, this study also provides empirical evidence for the mediating role of critical thinking disposition in the relationship between task difficulty and writing performance by establishing the structural equation model. These findings highlight the crucial role of critical thinking disposition in navigating task difficulty and achieving successful writing performance. This underscores the importance of cultivating critical thinking dispositions in EFL learners through targeted instruction and carefully designed classroom writing instruction.

By examining these complex interrelationships within a Chinese context, using data from a large-scale college English test, this study offers valuable insights for language educators and assessment developers. These findings can inform the design of writing tasks, the sequencing of instruction, and the development of assessment tools that promote both cognitive and affective engagement in EFL writing. Differentiating writing tasks across semesters is crucial for effective college English instruction. Ultimately, this research contributed to a more nuanced understanding of the factors that shape writing performance and provides practical recommendations for enhancing writing pedagogy and assessment based on Robinson’s componential framework [7]. These findings offer practical insights for instructors on fostering critical thinking in writing instruction.

## Supporting information

S1 Data Dataset containing writing test results from 201 college students. The file includes participants’ basic information (gender, major), scores from three raters, students’ writing performance scores, and their responses to task difficulty and critical thinking disposition. The critical thinking disposition section comprises 70 questions across 7 dimensions (e.g., Dimension 1 includes questions D11-D10, and so on). (XLSX)
